# Molecular Method for Sex Identification of Half-Smooth Tongue Sole (*Cynoglossus semilaevis*) Using a Novel Sex-Linked Microsatellite Marker

**DOI:** 10.3390/ijms150712952

**Published:** 2014-07-22

**Authors:** Xiaolin Liao, Genbo Xu, Song-Lin Chen

**Affiliations:** 1 Key Laboratory for Sustainable Development of Marine Fisheries, Ministry of Agriculture, Yellow Sea Fisheries Research Institute, Chinese Academy of Fishery Sciences, Qingdao 266071, China; E-Mails: xliao@mail.ihe.ac.cn (X.L.); eros1104@126.com (G.X.); 2 Key Laboratory of Ecological Impacts of Hydraulic-Projects and Restoration of Aquatic Ecosystem of Ministry of Water Resources, Institute of Hydroecology, Ministry of Water Resources and Chinese Academy of Sciences, Wuhan 430079, China

**Keywords:** *Cynoglossus semilaevis*, sex identification, sex-linked microsatellite marker (SLM), female-associated allele (FAA), multiplex PCR

## Abstract

Half-smooth tongue sole (*Cynoglossus semilaevis*) is one of the most important flatfish species for aquaculture in China. To produce a monosex population, we attempted to develop a marker-assisted sex control technique in this sexually size dimorphic fish. In this study, we identified a co-dominant sex-linked marker (*i.e.*, CyseSLM) by screening genomic microsatellites and further developed a novel molecular method for sex identification in the tongue sole. CyseSLM has a sequence similarity of 73%–75% with stickleback, medaka, Fugu and Tetraodon. At this locus, two alleles (*i.e.*, A244 and A234) were amplified from 119 tongue sole individuals with primer pairs CyseSLM-F1 and CyseSLM-R. Allele A244 was present in all individuals, while allele A234 (female-associated allele, FAA) was mostly present in females with exceptions in four male individuals. Compared with the sequence of A244, A234 has a 10-bp deletion and 28 SNPs. A specific primer (CyseSLM-F2) was then designed based on the A234 sequence, which amplified a 204 bp fragment in all females and four males with primer CyseSLM-R. A time-efficient multiplex PCR program was developed using primers CyseSLM-F2, CyseSLM-R and the newly designed primer CyseSLM-F3. The multiplex PCR products with co-dominant pattern could be detected by agarose gel electrophoresis, which accurately identified the genetic sex of the tongue sole. Therefore, we have developed a rapid and reliable method for sex identification in tongue sole with a newly identified sex-linked microsatellite marker.

## 1. Introduction

The half-smooth tongue sole (*Cynoglossus semilaevis*), belonging to Cynoglossidae in the Pleuronectiformes, is a large, commercially important, left-eyed marine flatfish species mainly inhabiting the Chinese coastal waters. Tongue sole has some interesting characteristics, such as having sex chromosomes with a ZZ/ZW sex determination system, and exhibiting sexual size dimorphism in favor of females [1,2,3,4,5,6]. These characteristics suggest that tongue sole has important values for monosex breeding as well as utility as a model for studying the mechanisms of sex determination [7]. Due to their high commercial value, tongue sole has been selected as an ideal species for aquaculture in China. However, the difficulty of attaining market size in aquaculture lowers the commercial value of male fish. Males are usually discarded after their sex is determined. Therefore, reducing the ratio of male to female or producing all-female stocks has significant economic implications in tongue sole aquaculture. It is necessary to establish super-female (WW) to produce all-female stock, as super-females could cross with normal males (ZZ) to produce all female (ZW) fry. Identification of super-female (WW) individuals is, therefore, of great value in establishment of a sustainable and economic means of producing an all-female tongue sole stock [2].

Sex-specific DNA markers can serve as efficient tools for molecular sex identification in fish production [8]. In tongue sole, some dominant sex-linked DNA markers have been developed using AFLP technology [2]. A tentative experiment has been conducted to produce high ratio of female fish by combining sex reversal with molecular marker-assisted sex control technique [1]. In that study, female-specific markers were successfully applied to identify neo-males (phenotypic males with female genotype) from normal males [2]. However, these dominant markers did not have the ability to distinguish the super-female (WW) from the normal female (ZW) within the tongue sole neo-male progeny. Therefore, it calls for sex-linked co-dominant markers (e.g., microsatellite DNA markers), which can identify the WW tongue sole and assist in developing a rapid and reliable method of molecular sexing and producing monosex stocks in tongue sole [3].

In the present study, we identified and characterized a sex-linked microsatellite marker and then developed a rapid and reliable molecular method for sex identification, which would be important for marker-assisted sex control breeding in tongue sole.

## 2. Results and Discussion

**Figure 1:**
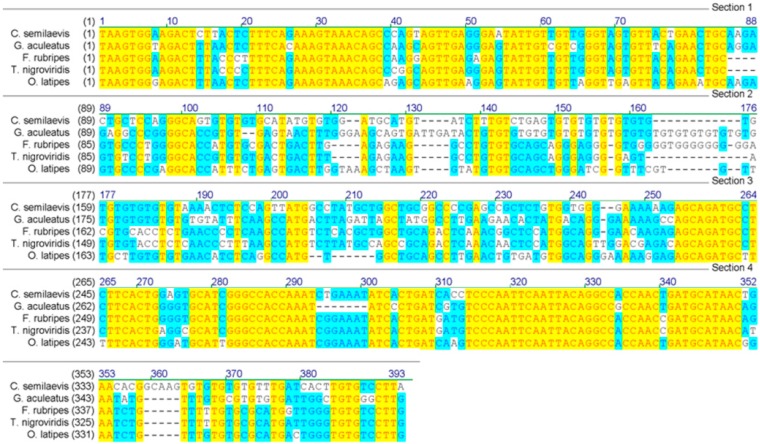
Alignment of tongue sole (*C. semileavis*) CyseSLM sequence with putative homologue sequences from stickleback (*G. aculeatus*.), medaka (*O. latipes*.), Fugu (*F. rubripes*.) and Tetraodon (*T. nigroviridis*).

We screened 26 microsatellite markers in 32 individuals (16 of each sex), and found that marker Cyse195 was monomorphic in males but polymorphic in females with two alleles. Another 87 individuals (44 females and 43 males) were used to further confirm this sex-linked marker. The microsatellite marker, Cyse195, was named as CyseSLM. A 373-bp-long sequence of CyseSLM with GT-repeat motif was deposited into GenBank (accession number: EU907064). Several putative homologue sequences were found by GenBank BLAST. The sequence of CyseSLM has a similarity of 73%–75% to the sequences from stickleback (*Gasterosteus aculeatus*, AC182809), medaka (*Oryzias latipes*, BAAF04020626 and BAAE01158496), Fugu (*Fugu rubripes*, CAAB01000203) and Tetraodon (*Tetraodon nigroviridis*, CAAE01014993) (Figure 1). Interestingly, the putative homologue sequence from stickleback also contained a microsatellite with the same repeat motif (Figure 1). The result demonstrated that this locus was comparatively conserved across more distantly related fish taxa. It also suggested that CyseSLM might be a part of a gene or tightly linked to a gene, therefore it has a potential use for cloning sex-related genes.

**Figure 2:**
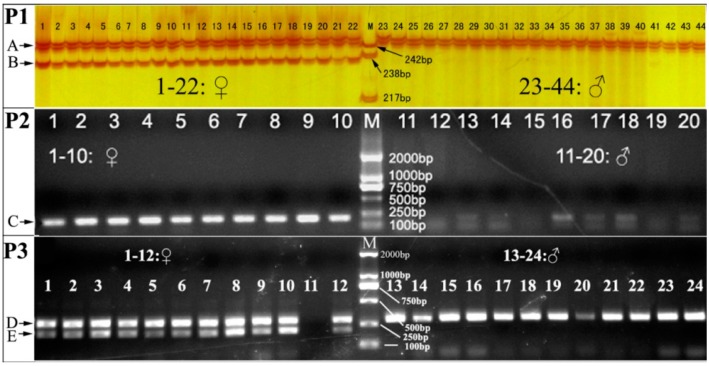
Electrophoresis pattern of the sex-linked microsatellite marker CyseSLM. In P1, amplified fragments using primers CyseSLM-F1 and CyseSLM-R; A and B are alleles A244 and A234, respectively; M: pBR322 DNA/*Msp* I molecular weight marker; In P2, amplified fragment using primers CyseSLM-F2 and CyseSLM-R; C is 204 bp amplicon; M: D2000 molecular weight marker; In P3, amplified fragments of multiplex PCR using primers CyseSLM-F2, CyseSLM-F3 and CyseSLM-R; D and E are 280 and 290 bp complex amplicon and 204 bp amplicon; M: D2000 molecular weight marker; DNA of female individual 11 was absent.

**Figure 3:**
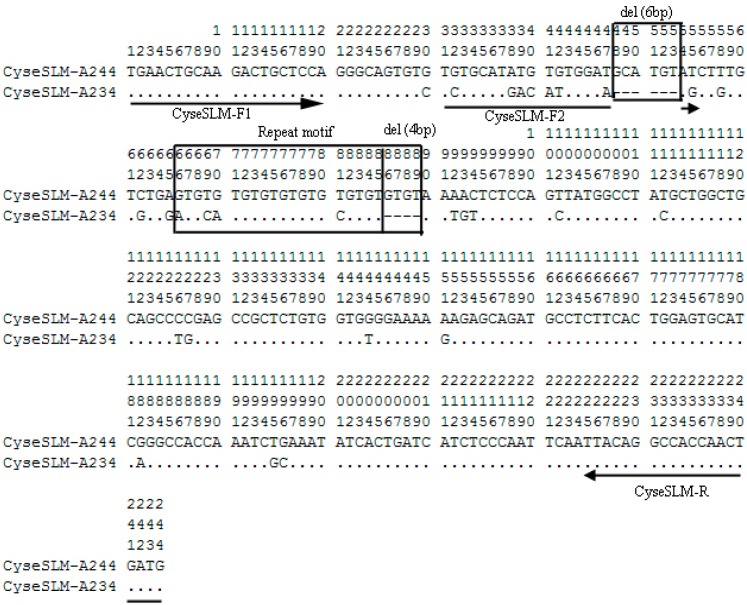
Alignment results and sequence variations between A234 and A244. Dots indicate identical bases, dashes indicate deletions.

Only two alleles (244 and 234 bp in size, identified as A244 and A234, respectively) were amplified in 119 individuals (60 females and 59 males) using primers CyseSLM-F1 and CyseSLM-R (Table 1). A244 was present in all individuals, while A234 was present in all females and only four males (Figure 2). The four males identified with A234 were neo-males [1]. This could be explained by the natural sex reversal at this developmental stage in the tongue sole rearing population [9]. The allele A234 could be female-associated allele (FAA), confirming that tongue sole adopts a ZZ/ZW sex-determining system [4,5,6,7]. Given the female heterogamety in tongue sole, the FAA A234 and A244 might be located on the W and Z chromosome, respectively. The sequences of alleles A234 and A244 were then deposited into GenBank (JF831036; JF831037) Compared to A244, A234 has a 10-bp deletion, of which 6-bp was located in the 5' flanking region and 4-bp of two GT copies in the repeat motif region. In addition, there were 28 single site mutations (SNPs) between A234 and A244 (Figure 3). The allelic variation in CyseSLM could also be used as a diagnostic tool for sex identification, so we designed a new forward primer (CyseSLM-F2) that crossed the 6-bp deletion region of the A234 sequence (Figure 3). A 204 bp fragment in all females and neo-males was amplified with CyseSLM-F2 and reverse primer CyseSLM-R (Table 1; Figure 2). The products of this allele-specific assay could then be directly detected by agarose gel electrophoresis.

**Table 1:** The primer combination for amplifying a sex-linked locus in *Cynoglossus semilaevis*.

PCR Name	Primer Names	Primer Sequences (5'—3')	Anneal Temp. (°C)	Expected Products Size (bp)	Primer Concentration (μM)	PCR Products Electrophoresis Medium
P1	CyseSLM-F1	tgaactgcaagactgctcca	61.5	234 (in female)	0.1	Polyacrylamide gel
	CyseSLM-R	catcagttggtggcctgtaa		244 (in male and female)	0.1	
P2	CyseSLM-F2	tctgcatgacattggaaag	58	204 (in female)	0.1	Agarose gel
	CyseSLM-R	catcagttggtggcctgtaa			0.1	
P3	CyseSLM-F2	tctgcatgacattggaaag	60	204 (in female)	0.1	Agarose gel
	CyseSLM-F3	cagcccagtagttgagggaat		280 (in female)	0.02	
	CyseSLM-R	catcagttggtggcctgtaa		290 (in male and female)	0.12	

Polyacrylamide gel electrophoresis with silver staining was used to detect the PCR products of CyseSLM, but it is complicated and time-consuming. We threfore simplified the process by detecting the co-dominant pattern of PCR amplicons of CyseSLM using agarose gel eletrophoresis. A new forward primer (CyseSLM-F3) was designed based on 5' flanking region of CyseSLM sequence (EU907064), which amplified the expected 280- and 290-bp fragments in females and 290-bp fragment in males with the reverse primer CyseSLM-R, respectively. Subsequently, we developed a multiplex PCR program with a primers combination of CyseSLM-F2, CyseSLM-F3 and CyseSLM-R primer (Table 1). In the multiplex PCR system, the concentration of CyseSLM-F3 was only one fifth of that of CyseSLM-F2 (Table 1) due to competitive amplification of primer pair CyseSLM-F3/CyseSLM-R against that of primer pair CyseSLM-F2/CyseSLM-R. The 280- and 290-bp fragments could not be clearly separated in agarose gels of low resolution, so there were two bands in females (280- and 290-bp complex amplicons and 204-bp amplicon) but only one band in males (290-bp amplicon). Figure 2 shows a co-dominant pattern of the multiplex PCR amplicons in agarose gel. Overall, the sex-linked marker CyseSLM was successfully converted to a co-dominant marker that could be detected in agarose gel. Such a rapid, reliable and low-cost method as developed in this study is useful in the molecular identification of super-female and production of all-female tongue sole stocks in the future.

In the present study, we did not find indication of recombination between the sex-determination locus and the locus CyseSLM. The distinct allelic divergence at CyseSLM between the sex chromosomes could be caused by the suppression of recombination between these chromosomes. The sex chromosomes of tongue sole (*i.e.*, Z and W chromosomes) are highly different from each other [1,5,6,7]. The accumulations of repetitive DNA sequences lead to morphologically differentiated sex chromosomes and gave rise to chromosome heterochromatization, and then recombination between sex chromosomes was suppressed [10,11]. As repetitive DNA sequences, microsatellites would be of great potential use in the development of sex-linked markers in fish species. Recently, there were several reports on sex-linked microsatellite markers in different fish species [11,12,13].

## 3. Experimental Section

The tongue sole fish used in this study, as part of the broodstock population, was collected from Laizhou Mingbo Aquatic Company, Shandong, China. Their phenotypic sex was confirmed during artificial reproduction. DNA samples were extracted from fin tips using the traditional phenol-chloroform procedure. In total, 26 polymorphic microsatellite markers (Cyse61, 62, 67, 70, 72, 79, 90, 93, 99, 113, 134, 136, 145, 147, 149, 151, 159, 160, 176, 188, 195, 198, 209, 215, 217, 232) were employed to screen sex-associated markers. These markers were initially developed for constructing a genetic linkage map, but they were monomorphic or had significant segregation distortion in the mapping family [14]. Firstly, 32 tongue sole individuals (16 of each sex) were used to test the difference of the microsatellite loci between females and males. Once a given marker was considered as a putative sex-linked one, another 87 individuals (44 females and 43 males) were genotyped to confirm it. PCR amplified products were separated in 6% denaturing polyacrylamide gel using silver staining.

The purified PCR products of sex-linked marker CyseSLM on six individuals (three of each sex) were ligated into a pMD18-T vector (Takara, Dalian, China) and transformed into DH5α *E. coli* competent cells (Invitrogen, Beijing, China). Positive clones (2–8 for each individual) were sequenced on an ABI3730 sequencer. The sequences were aligned by MEGA 4.0 software (Tempe, AZ, USA). According to the results of alignment, the primer CyseSLM-F2 and CyseSLM-F3 were designed using online software PRIMER3 (Cambridge, MA, USA). 

## 4. Conclusions

We characterized a novel sex-linked microsatellite marker CyseSLM in tongue sole and further developed a rapid and reliable molecular method for sex identification based on multiplex PCR method. This method provides a useful tool for controlling sex and cloning sex-related genes in the tongue sole.
